# Comprehension of Generalized Conversational Implicatures by Children With and Without Autism Spectrum Disorder

**DOI:** 10.3389/fpsyg.2018.00272

**Published:** 2018-03-13

**Authors:** Gemma Pastor-Cerezuela, Juan C. Tordera Yllescas, Francisco González-Sala, Maite Montagut-Asunción, María-Inmaculada Fernández-Andrés

**Affiliations:** ^1^Basic Psychology, Facultad de Psicología, Universitat de València, Valencia, Spain; ^2^Teaching of Language and Literature, Facultad de Magisterio, Universitat de València, Valencia, Spain; ^3^Developmental and Educational Psychology, Facultad de Psicología, Universitat de València, Valencia, Spain

**Keywords:** non-literal meaning, implicatures, Levinson model, children, autism spectrum disorder

## Abstract

This study evaluates the comprehension of generalized conversational implicatures (GCI) in children with and without autism spectrum disorder (ASD), using a GCI test constructed based on the Levinson model, which distinguishes between three types of implicatures: type Q (or scalar: “what is not referred to does not occur”); type I (“by default, it is not necessary to say what can be assumed”); and type M (“if someone is expressing something in a not very simple or marked way, it is because s/he is describing a situation that is not very typical, frequent, or prototypical”). In addition to the ASD group (*n* = 22), two comparison groups were utilized: a group matched on chronological age with the ASD group, but with a higher linguistic age (TCD group, *n* = 22), and a group matched on linguistic age with the ASD group, but with a lower chronological age (TLD group, *n* = 22). In all cases, linguistic age was assessed with the Peabody test. The performance of the three groups on the GCI test was compared (overall and on each type of implicature), and performance on the three types of implicature was compared within each group. The ASD group obtained worse performance than the other two groups, both overall and for each implicature type, without also obtaining differences in performance on the three implicature types. The TCD group obtained better performance than the TLD group on overall performance, but not on each implicature type, and both groups obtained lower performance on the type M heuristics than on the type I. Based on these results, the children with ASD in our study presented limitations in the comprehension of the three types of GCI, but it was not possible to obtain evidence for an inferential continuum of the three types of GCI. However, in the two typical development groups, this evidence was obtained, leading us to propose an inferential continuum model based on the different levels of dependence on the context of each of the three types of implicatures, with type M implicatures being more contextually dependent.

## Introduction

Autism spectrum disorder (ASD) is a disorder included in the category of “neurodevelopmental disorders” in the Diagnostic and Statistical Manual of Mental Disorders (DSM-5; American Psychiatric Association, 2013). It involves the presence of persistent difficulties in communication and social interaction across multiple contexts, along with restrictive, repetitive, and stereotypical patterns of behavior, activities, and interests. The symptoms must be present in the first phases of development, they must produce significant clinical impairment in various areas of functioning, and they cannot be explained by the presence of an intellectual disability. To make the ASD diagnosis, it is necessary to specify whether there is another associated condition or disorder, such as a language impairment.

Deficits in verbal language and the heterogeneity and variability of the linguistic abilities of people with ASD have become widely recognized and researched (Groen et al., 2008; Eigsti et al., 2011; Boucher, 2012). One type of linguistic limitation that usually appears in ASD is related to the comprehension of figurative language and non-literal meaning (Tager-Flusberg, 1985, 1991; Happé, 1993, 1995; Hill, 2004; Norbury, 2005; Hill and Bird, 2006; Roundblad and Annaz, 2010).

### Non-literal meaning and implicatures

Non-literal meaning refers to non-explicit meaning, or what is not said. It is a concept that includes many others, such as entailments, presumptions, and implicatures. Among them, the implicatures have received special attention among pragmatists. According to Grice (1975), in implicatures, social rules are in play that describe the characteristics of the ideal communicative exchange and determine the expectations of rational speakers about the linguistic behavior of the other speakers. When these rules appear to be violated, it is necessary to make inferences (conversational implicatures) in order to guarantee the fulfillment of these maxims. Among the conversational implicatures, we can distinguish between generalized and particularized implicatures.

Generalized conversational implicatures (GCI) are inferences that refer to the non-explicit meaning that occurs by default in any type of context (Grice, 1975). It is information that is inferred in a prototypical way, as long as there is no specific information that denies or contradicts it. By contrast, particularized conversational implicatures (PCI), also called *ad-hoc* implicatures, are closely linked to specific or particular contexts; that is, the success of these inferences is linked to knowledge about very specific contextual information. PCI and GCI have one defining characteristic, compared to other non-explicit meanings such as entailments or conventional implicatures. They are cancellable; that is, if the context changes or is enriched or modified, conversational implicatures can disappear. In order to clarify these concepts, examples (1) and (2) are proposed, where a PCI and a GCI appear, respectively:

1. (Speaker B, who is short, does not like to go out with tall women, and speaker A knows this).
  -Speaker A: Do you want Ana's telephone number so you can go out with her?
  -Speaker B: Ana is quite tall.
  PCI >> Speaker B does not want speaker A to give him Ana's telephone number to go out with her.
2. Juan has three children.
  GCI >> Juan has exactly three children, not more or less.

In the example (1), the prior knowledge that speaker B does not like tall women is what allows us to reach PCI, but if this prior context changed (for example, if B liked tall women), the inferred PCI could change. However, in example (2), we do not need any specific prior information to reach GCI. By default, any speaker would tend to infer that Juan has exactly three children and no more, and as long as no additional information is provided to contradict this (for example, *Well, and he has a fourth child who was adopted 5 years ago*), the GCI is maintained by default.

Grice's theoretical paradigm was later continued and partially modified by authors such as Levinson (2000) in the Theory of Generalized Conversational Implicatures. This theory defends the existence of a linguistically coded meaning and the distinction between three heuristics that make it possible to interpret the different GCI: the Q heuristic, the I heuristic, and the M heuristic.

The Q heuristic, “What isn't said, isn't,” is based on Grice's premise “Make your contribution as informative as required.” It establishes that what is not referred to does not occur. This heuristic is also called scalar implicature, based on the idea that there are elements that conform an informative scale ranging from the weakest element (e.g., *some*) to the strongest (e.g., *all*). Therefore, if the speaker decides to use the weakest element on the scale, it is because he/she considers that the strongest element is not true (therefore, from *some, not all* can be inferred). Thus, based on the sentence “Some of the guests came to Maria's party,” it can be inferred that “not all of the guests that Maria expected came.” This is an example of GCI legitimized by the Q Heuristic (or scalar implicature).

The I Heuristic, “What is simply described is stereotypically exemplified,” is based on Grice's premise “Do not make your contribution more informative than is required.” It establishes that, by default, it is not necessary to say what can be assumed. Thus, from the sentence “Pedro and Maria bought a flat,” it would have to be inferred that “Pedro and Maria bought one flat together.” This is an example of GCI legitimized by the I Heuristic.

The M Heuristic, “What's said in an abnormal way, isn't normal” or “a marked message indicates a marked situation,” is based on Grice's premises “Avoid obscurity of expression” and “Be brief (avoid unnecessary prolixity).” It establishes that, if someone is expressing something in a not very simple or marked way, it is because s/he is describing a situation that is not very typical, frequent, or prototypical. Thus, the sentence “Antonio stopped the car” leads to the inference that “Antonio did it in a stereotypical way: with his foot on the brake pedal.” However, the sentence “Antonio made the car stop” leads to inferring that “Antonio stopped it in an unconventional way.” This latter case would be an example of GCI legitimized by the M Heuristic.

Two theoretical proposals explain the way implicatures are processed (Degen and Tanenhaus, 2015): (1) the Literal-First hypothesis, which argues that the inferred meaning has to subsequently be added to the literal meaning; and (2) the Constraint-Based framework, which denies this sequential nature of the processing.

The former proposal (literal-first hypothesis) includes two lines of research. The first (Levinson, 2000; Chierchia, 2004) assumes that processing GCI does not involve a considerable additional processing cost. GCI are computed immediately and with no effort, given that they are inferences that occur from below; thus, unlike PCI, in GCI, the contextual information would not be relevant to their resolution. The second line of research, however, considers that all implicatures (GCI and PCI) require some type of additional time and cognitive resources (Huang et al., 2010).

The latter proposal (the Constraint-Based framework), defended by Degen and Tanenhaus (2015), states that inferences do involve a processing cost, but that this cost will vary depending on the relevance of the context. Thus, if the context provides little or no help in the interpretation, the cognitive cost will be greater. Therefore, it is a question of how relevant the context can be in determining the processing cost.

It should be pointed out that, to the extent that the present study is pragmatic, the pragmatic aspect was difficult to delimit within Linguistics itself. Thus, for example, Grice (1975) interprets his maxims as social rules. Sperber and Wilson (1986) reduce the pragmatic aspect to principles of cognitive processing. Levinson (2000) addresses GCI and considers them to be a clearly linguistic element (neither social nor cognitive). Other authors, such as López-García (1989) or Escandell-Vidal (2011), defend postures of consensus. López-García (1989) defends the idea that Pragmatics is a borderline discipline between Internal Linguistics (Syntax, Morphology) and External Linguistics (Sociolinguistics, Psycholinguistics). In addition, Escandell-Vidal (2011) talks about a cognitive Pragmatics (which does not study the sociocultural rules of a society) and a social Pragmatics (which would be characterized by just the opposite, and would study events such as courtesy, indirect speaking acts in a social context, etc.). Our theoretical position comes closer to the position of these two latter authors, and we believe it is advisable to distinguish between a more linguistic Pragmatics (more linked to formal knowledge, that is, morphology, syntax, and lexicon) and another more external Pragmatics (where social and cognitive skills are necessary).

### Development of implicatures in children with and without ASD

The study on scalar implicatures (type Q) carried out by Pouscoulous et al. (2007) with a population of children and adults concluded that children are not able to make scalar implicatures in the same way adults do until the age of seven, although the process begins at the age of four. Moreover, they pointed out that the lexical complexity (e.g., *some* vs. *certain*) and the introduction of the negation (e.g., *some* vs. *none*; *all* vs. *not all*) could be factors that impede the consolidation of inference comprehension.

Other studies state that acquisition of the inferential capacity is achieved at quite early ages (Papafragou and Tantalou, 2004; Stiller et al., 2015; Yoon et al., 2015; Skordos and Papafragou, 2016). Thus, PCI or *ad-hoc* implicatures can be acquired from the age of three with the help of prosodic cues, so that at 4 years old, this capacity is fairly consolidated (Yoon et al., 2015). Some authors indicate that cognitive abilities such as the Theory of Mind (ToM) can offer an explanation, but only partially, about inferential development (specifically PCI). Thus, Bosco and Gabbatore (2017) indicated that first-order ToM has a causal role in explaining children's performance in handling sincere and deceitful speech acts, but not irony. In addition, Angeleri and Airenti (2013) concluded that the comprehension of jokes and ironies in children from 3 to 6 years old does not require completely developed ToM skills, whereas the production of judgements about communicative acts is a ToM task. In the comprehension of the communicative intention, the children, like the adults, depend on other factors, such as their familiarity with the situation and accessibility of the knowledge that forms the background of the communicative act. In the case of GCI, Eiteljörge et al. (2016) state that as soon as children acquire the indefinite pronoun *someone*, at about 3 years old, they start to make scalar implicatures. It is also true that recent studies, like the one by Sullivan et al. (2017), relativize the inferential capacity of children and consider that other capacities (e.g., mutual exclusivity) allow them to perform scalar implicatures.

With regard to the inferential capacity in ASD, the different studies carried out (e.g., Pijnacker et al., 2009; Chevallier et al., 2010; Whyte and Nelson, 2015) reached the conclusion that lexical competence and syntactic competence are good indicators of pragmatic competence, which is undoubtedly involved in the inferential capacity (especially in GCI). However, in the pragmatic competence, other cognitive skills are involved, in addition to the syntactic-lexical ones, such as skills of interpreting the intentions and emotions of others, the use of language for multiple functions, the expression of internal states, and adjusting to the speaker's social status. Some more complex skills can even be involved, such as calibrating the information a speaker needs and taking into account what s/he already knows. According to Monfort et al. (2004), these skills, related to communication and social interaction, confer a socio-cognitive use to language and generally present limitations in people with ASD. However, Kissine (2016) considers that some of these abilities, which pertain to theory of mind (ToM), would not be necessary for pragmatic processing. In any case, this socio-cognitive use of language (Monfort et al., 2004) has early indicators (or precursors) that date back to pre-verbal stages in the case of typical development, but are compromised in autistic development (Bopp and Mirenda, 2011; Eigsti et al., 2011; Boucher, 2012). The difference in the presence of these pre-verbal indicators in children with and without ASD could contribute, partly, to differences in later pragmatic competence (Monfort et al., 2004).

In fact, regarding the comparison of ASD and typical development, Whyte and Nelson (2015) indicate that children with ASD develop the inferential capacity of PCI at a slower rhythm than their peers with typical development. However, in the studies by Pijnacker et al. (2009) with adults, and Chevallier et al. (2010) with adolescents, the authors pointed out that there were no noteworthy differences in the comprehension of scalar implicatures between people with and without ASD. Thus, it is possible that the acquisition of inferential abilities is slower in ASD than in typical development during the childhood stage, but later, when children with ASD reach the stages of adolescence and adulthood, these differences become diluted in most cases.

### Objectives of the present study

The main objective of the present study was to evaluate GCI comprehension by children with and without ASD. To do so, a GCI test was constructed, based on the model by Levinson (2000). This theory was chosen because this theoretical model is constructed *ex profeso* to explain the idiosyncrasies of GCI. In addition, it is a valid model to explain the transition between the semantic and the pragmatic on an inferential continuum (entailment > presupposition > conventional implicature > conversational implicature: GCI > PCI). On this inferential continuum, entailment is the non-explicit content obtained independently from the context and more linked to the lexical meaning of the elements, whereas, on the other extreme, PCI can only be validated according to the specific context given. In the middle, there are other concepts that would be more or less dependent on the context.

This study is focused on GCI, a type of implicature that involves constructs that are not exclusively semantic or pragmatic (Levinson, 2000), but instead include both aspects. Thus, good performance on this type of implicature requires both syntactic-lexical and pragmatic competencies. In the present study, in order to compare the GCI performance of children with and without ASD, in addition to the ASD group, two comparison groups were used: a group matched on chronological age with the ASD group, but with a higher linguistic age (TCD group), and a group matched on linguistic age with the ASD group, but with a lower chronological age (TLD group). In all cases, the linguistic age was evaluated with the Peabody test (Dunn et al., 2006).

The specific objectives of the present study were the following:

Objective. 1. Determine whether there were differences among the three groups of subjects on the overall performance obtained on the GCI test.
Objective. 2. Determine whether there were differences among the three groups of subjects on the performance obtained on each of the three types of implicatures (Q, I, and M) included in the GCI test.
Objective. 3. Determine whether, within each group, there were differences in the performance obtained on the three types of implicatures (Q, I, and M) included in the GCI test.

As far as we know, the Levinson model has not been previously applied to research on ASD or in relation to a proposed inferential continuum, and this is where our study makes an original contribution to the literature.

## Materials and methods

### Participants

The participants in the present study consisted of 66 elementary school children from 6 to 13 years old with a non-verbal IQ above 85. The 66 children were divided into three groups. The ASD Group (*n* = 22) was composed of 18 males and 4 females with a mean chronological age of 10.92 years (*SD* = 1.19) and a mean linguistic age of 8.28 years (*SD* = 1.88) on the Peabody test (Dunn et al., 2006). The TCD Group (*n* = 22) was composed of 16 males and 6 females with a mean chronological age of 10.89 years (*SD* = 1.02) and a mean linguistic age of 11.48 years (*SD* = 1.55) on the Peabody test. This was a group of children with typical development matched on chronological age with the ASD Group. The TLD Group (*n* = 22) was composed of 16 males and 6 females with a mean chronological age of 8.35 years (*SD* = 0.32) and a mean linguistic age of 8.64 years (*SD* = 2.02) on the Peabody test. This was a group of children with typical development matched on linguistic age with the ASD Group.

Children in the ASD Group had a clinical diagnosis of ASD, according to the DSM-IV-TR criteria (American Psychiatric Association, 2000), and they met the diagnostic criteria for level 2 of the DSM-5 (American Psychiatric Association, 2013). They had been diagnosed by neuropediatric services from different hospitals in the national health system. These neuropediatric services were responsible for checking compliance with these diagnostic criteria. They referred the children who met the diagnostic criteria to early care units, where the diagnosis was confirmed using more specific instruments, such as the Autism Diagnostic Observation Schedule (ADOS), which was applied by specialized psychologists who had official accreditation to use this instrument. Moreover, all of them obtained an Autism Index score ≥85 on the Gilliam Autism Rating Scale, Second Edition (GARS-2), indicating a high likelihood of the disorder (Gilliam, 2006). The scores on the GARS-2 ranged from 85 to 135 (*M* = 95.25, *SD* = 8.30). The children in the ASD Group were attending schools with specific classrooms in which the Treatment and Education of Autistic and Related Communication Handicapped Children (TEACCH) methodology was carried out. These are integrated classrooms included in regular state schools in Valencia (Spain) where students with disorders affecting language and communication are enrolled. The children in the two Comparison Groups were children with typical development, without any clinical diagnosis, who attended the same schools as the ASD Group, but in the regular modality.

No statistically significant differences were found among the three groups of children on gender (χ^2^ = 0.518, *p* = 0.472). With regard to the linguistic age, there were significant differences [*F*_(2, 63)_ = 20.01, *p* < 0.001, η^2^_*p*_ = 0.389], as it was higher in the TCD group than in the ASD and TLD groups, with no differences between the latter two. In the case of chronological age, there were significant differences [*F*_(2, 63)_ = 20.64, *p* < 0.001, η^2^_*p*_ = 0.396], as it was lower in the TLD group than in the ASD and TCD groups, with no differences between the latter two. Thus, the ASD group was matched with the TCD group on chronological age and with the TLD group on linguistic age.

### Ethics statement

This study is part of an investigation that was approved and funded by the University of Valencia, and the Valencian government. The research had the official and written authorization of the General Direction and School Management (Valencia Education, Training and Employment Department). Moreover, written authorization to carry out the research was obtained from the participating schools, and the children's parents.

### Procedures

Each child's performance on the Peabody test and the GCI test was individually evaluated in a noise- and distraction-free office. In all cases, the tasks were administered in the same order (first: the Peabody test, and second: the GCI test). Information about autism symptoms was obtained from the GARS-2 through an interview with the parents of the ASD Group.

### Instruments

*Generalized conversational implicature test (GCI test)*. This instrument, which was designed in computerized format, includes a total of 15 items that can be grouped in three different implicatures: type Q or scalar implicatures (“What isn't said, isn't”); type I implicatures (“What is simply described is stereotypically exemplified”); and type M implicatures (“What's said in an abnormal way, isn't normal; or a marked message indicates a marked situation”). The instrument includes five items for each type of implicature. Each item is composed of a statement and three possible response options. The subject had to point to or indicate which of the three response options fit best or corresponded to the sentence in the item. A full translation of the original Spanish version of the instrument can be found in Appendix 1 in Supplementary Material. For example, for the statement: “Some guests came to Maria's party”, the response options are: (a) All the people Maria invited came; (b) Not all the guests Maria expected came; and (c) Exactly three guests came. Three experts in Linguistics independently performed the assignment of the statements in the items to the three types of implicatures (Q, I, and M). The test items were presented randomly. For each subject, the number of correct answers was recorded (the total number and those obtained on each type of implicature) and the time taken to perform the test. Previously, training on the task was provided, administering various items that were different from those on the test, in order to make sure the subject understood the task he/she was going to do.

### Data analysis

Data analyses were performed using the SPSS version 24 statistical package. For the first objective, two ANOVAs were conducted (one for the number of correct answers, and the other for the response time) to compare the overall performance of the three groups of subjects on the GCI test. For the second objective, three ANOVAs were conducted (one for each type of implicature: Q, I, and M) to compare the performance (number of correct answers) of the three groups of subjects. For the third objective, three repeated-measures multivariate analysis were conducted (one for each group of subjects) to compare the performance (number of correct answers) within each group on the three types of implicatures (Q, I, and M). In all cases, confirmation ANOVAs and pairwise comparisons were also carried out. Furthermore, in order to control the probability of type I error, the Bonferroni correction was used, establishing the significance level at less than or equal to 0.016. Effect sizes were calculated using partial η^2^ values, according to Cohen (1988): η^2^ = 0.06, small effect size; η^2^ = 0.06 to 0.14, medium; η^2^ = 0.14, large.

## Results

### Objective 1. group differences in the overall performance on the GCI test

Table 1 presents the results of the ANOVAs conducted to compare the overall performance on the GCI test of the three groups of subjects and pairwise comparisons. The results showed statistically significant differences among the three groups. Thus, the TCD group showed the best performance on both the number of correct answers and the response time, and the ASD group showed the worst performance.

**Table 1:** Group differences in the overall performance (correct answers and response time) obtained on the GCI test.

**Measure/ group**		**ASD**	**TCD**	**TLD**	***F*_(2, 63)_**	***p***	**η^2^**	**Differences between groups**
Correct answers	M	7.18	12.45	10.27	33.65	0.001	0.517	ASD<TLD<TCD
	SD	2.97	1.26	1.83				
Response time	M	512	240.77	357.21	23.93	0.001	0.431	ASD>TLD>TCD
	SD	189.10	61.11	107.47				

*p ≤ 0.016. Bonferroni correction of critical p-values when performing multiple comparisons*.

### Objective 2. group differences in the performance obtained on each type of implicature (Q, I, and M) included in the GCI test

Table 2 presents the results of the ANOVAs conducted to compare the performance of the three groups of subjects on each type of implicature and pairwise comparisons. On all three implicature types, the ASD group presented worse performance on the number of correct answers than the two comparison groups. For the two comparison groups, although the data indicate that the TCD group obtained more correct answers than the TLD group on all three implicature types, these differences did not reach statistical significance in any case.

**Table 2:** Group differences in the performance (correct answers) on each type of implicature (Q, I, and M) included in the GCI test.

**Group/GCI**		**ASD**	**TCD**	**TLD**	***F*_(2, 63)_**	***p***	**η^2^**	**Differences between groups**
Q	M	2.36	4.27	3.36	16.51	0.001	0.343	ASD<TCD; TLD
	SD	1.25	0.70	1.25				
I	M	2.81	4.59	4.00	16.95	0.001	0.349	ASD<TCD; TLD
	SD	1.46	0.59	0.81				
M	M	2.00	3.59	2.95	15.79	0.001	0.333	ASD<TCD; TLD
	SD	1.06	0.90	0.84				

*p ≤ 0.016. Bonferroni correction of critical p-values when performing multiple comparisons*.

### Objective 3. within-group differences in the performance on the three implicature types (Q, I, and M) included in the GCI test

The results of the repeated-measures multivariate analysis conducted to compare, within each group, the performance obtained on the three types of implicatures (Q, I, and M) showed that, in the ASD group, there were no statistically significant differences in the performance on the three types of implicatures after the application of the Bonferroni correction [Wilks' Lambda = 0.723, *F*_(2, 20)_ = 3.82, *p* = 0.039, ${\text{η}}_{p}^{2}$ = 0.277]. However, in the other two repeated-measures multivariate analyses, statistically significant differences were found in the TCD and TLD groups among the types of implicatures [TCD group: Wilks' Lambda = 0.576, *F*_(2, 20)_ = 7.37, *p* = 0.004, ${\text{η}}_{p}^{2}$ = 0.424; and TLD group: Wilks' Lambda = 0.541, *F*_(2, 20)_ = 8.31, *p* = 0.002, ${\text{η}}_{p}^{2}$ = 0.454]. Differences were found between the type I and M implicatures, with the performance of both groups being higher in the case of type I implicatures (see Table 3).

**Table 3:** Within-group differences in performance (correct answers) on the three types of implicatures (Q, I, and M).

**Group/GCI**		**Q**	**I**	**M**	***F*_(2, 43)_**	***p***	**η^2^**	**Differences between types of implicatures**
ASD	M	2.36	2.81	2.00	3.82	0.030	0.154	
	SD	1.25	1.46	1.06				
TCD	M	4.27	4.59	3.59	10.11	0.001	0.325	I>M
	SD	0.70	0.59	0.90				
TLD	M	3.36	4.00	2.95	6.66	0.003	0.241	I>M
	SD	1.25	0.81	0.84				

*p ≤ 0.016. Bonferroni correction of critical p-values when performing multiple comparisons*.

## Discussion

Most of the previous studies on GCI (Papafragou and Tantalou, 2004; Pouscoulous et al., 2007; Pijnacker et al., 2009; Chevallier et al., 2010; Stiller et al., 2015; Yoon et al., 2015; Skordos and Papafragou, 2016) had only focused on a subset of GCI, that is, scalar or type Q heuristic implicatures. However, Levinson's model (Levinson, 2000) includes other types of implicatures (type I and M heuristics) that had mainly been ignored. The contribution of the present study lies in discriminating between these three types of implicatures, which, in light of the results obtained, seem to be psycho-linguistically relevant.

The main objective of this study was to evaluate GCI comprehension in children with and without ASD, using a GCI test constructed on the basis of the Levinson's model (Levinson, 2000) of Generalized Conversational Implicatures, which distinguishes between three types of implicatures: Q (or scalar) heuristics, I heuristics, and M heuristics. In order to compare the GCI performance of children with and without ASD, in addition to the ASD group, we used two comparison groups: a group matched on chronological age with the ASD group, but with a higher linguistic age; and a group matched on linguistic age with the ASD group, but with a lower chronological age (TLD group).

The overall results obtained on the GCI test, both on correct answers and the total time taken to do it, showed that the best performance was obtained by the TCD group, followed by the TLD group, whereas the group with the worst overall performance was the ASD group. However, when comparing the performance of the three groups (correct answers) on each type of implicature separately, the ASD group performed worse than the two typical development groups, with no significant differences between the two typical development groups on any of the three types of implicatures. The group of children with ASD presented, therefore, greater deficits in the comprehension of inferential language (on the three types of GCI) than the two groups with typical development. This result would coincide with the findings of Whyte and Nelson (2015), who, although focusing on PCI, showed that children with ASD develop inferential capacity at a slower rhythm than their peers with typical development. Our hypothesis is that the limitations in the socio-cognitive component of language that are usually present in ASD (Monfort et al., 2004) could be the basis of this difference found between the ASD group and the two groups with typical development. As mentioned in the introduction, the pragmatic skills (involved in processing GCI) include a series of cognitive skills, including socio-cognitive comprehension skills, whose indicators (or precursors) generally begin to develop in early pre-verbal stages of children with typical development, but in a more limited and compromised way in children with ASD. In any case, more studies would be needed in this direction, also taking into account the recent research area indicating that some mentalist abilities might not be necessary for pragmatic processing (Kissine, 2016).

When we compared the performance (correct answers) on the three types of implicature within each group, no differences were found in the ASD group. One possible reason for this result could be the reduced number of items included in each type of implicature (only 5), which may have been insufficient to find performance differences between the three types of implicature in this group. It is also possible that all three types of implicatures were of such difficulty and complexity for the subjects in the ASD group that there was a sort of “floor effect” that did not yield differences. Therefore, if the ASD group presents important limitations in their inferential capacity, they might not obtain differences in the results for the three types of implicatures included in GCI. Thus, the idea that the inferential capacity lies on a continuum would not be supported (at least in ASD), contradicting results obtained from other studies on figurative language in ASD, such as those found by Melogno et al. (2012) on metaphors in high-functioning ASD. These authors indicated that the interpretation of the metaphor cannot be reduced to stagnant and monolithic criteria, but rather degrees of interpretation can be established (Melogno et al., 2012). In any case, although our results do not agree with the idea of an inferential continuum of GCI in the case of ASD, the preliminary nature of our study means that there is a need for other future studies on this topic.

However, when we compare the performance (correct answers) on the three types of implicatures within each of the two typical development groups, the results obtained support the idea of the GCI inferential continuum. Thus, the two typical development groups resolved the type M implicatures worse than the type I, with no differences in the result obtained for type Q implicatures. These results lead us to hypothesize that the type M and type I implicatures would be extremes on a continuum, where the children with typical development in our sample would have less difficulty with the I implicatures and greater difficulty with the M implicatures, whereas the Q implicature values would lie in the middle (I >> Q >> M). Thus, if the type I and type M implicatures are introduced, we cannot assume that GCI are acquired at the early age of 3 years, as Eiteljörge et al. (2016) pointed out. Instead, the process of consolidating the inferential capacity (especially, due to the incorporation of the M implicatures) would be a gradual and constant process.

In our opinion, the processing of sentences that require the application of the I and Q implicatures shares the fact that, to be interpreted, the weight of the development of the logical-semantic skills is greater than that of any other cognitive skill (whether proposed by the ToM, or by Weak Coherence Theory). The I implicature means that the preferred interpretation is the one most often associated with a term or set of terms (for example, given *if*, interpreting it by default as *if and only if*). The Q implicature means that, given the weak term, the strong term is denied (that is, given *some*, interpreting it as *not all*). Neither of the two cases require other skills, such as knowing the mental state of the speaker. By contrast, when the M implicature appears, which we view as a bridge/limit implicature between GCI and PCI, the logical-semantic skills are insufficient: the activation of the M implicature only determines that the situation described is not a usual or prototypical situation, but in no case does the implicature transmit which specific situation the statement is describing. In this case, the cognitive skills would be needed that are present in the processing of PCI, and that current studies in the field of ASD seem to identify in the ToM or in Weak Coherence Theory (Loukusa and Moilanen, 2009).

Therefore, the results obtained in the two typical development groups lead us to propose that the inferential capacity would not be an all or nothing process, but instead would lie on a continuum: I >> Q >> M. The type I implicatures would be easier to understand than type M implicatures, which would be the most difficult. This fact would be due to the greater or lesser contextual dependence accompanying the different types of implicatures, which coincides with the theoretical proposal defended by Degen and Tanenhaus (2015), the Constraint-Based framework. Thus, the more contextual help is needed to untangle the type of inference, the greater the processing cost (empirically verifiable by a longer response time and by making more errors), and the later this type of inference will be acquired. Moreover, the more contextually dependent the inference is, the more necessary the intervention in other cognitive skills. Consequently, a continuum can be established between linguistic pragmatics and social pragmatics (Kalandadze et al., 2016; Andrés-Roqueta and Katsos, 2017) or, as we prefer to emphasize, between a more linguistic pragmatics and a more socio-cognitive pragmatics. In this regard, the M implicature, which is the closest to PCI, would be the one most dependent on the context, and so its correct resolution would require contextual information, which could explain why these implicatures were more difficult than type I implicatures.

In sum, our proposal is that, in the case of children with typical development, the three different types of implicatures proposed by Levinson (2000) would have different degrees of processing difficulty, and this would be due to the degree of contextual dependence. The type I heuristics (which would be closest to conventional implicatures) would be less dependent on the context, and the most context-dependent would be the type M heuristics (which would be the closest to PCI).

With regard to the scalar, or type Q, implicatures, a logical system of relationships comes into play with numerous pairs of elements (e.g., *some* and *all*; *can* and *must*; *possible* and *necessary*, etc.), or at least they are obtained when the strong term has been negated by the weak term (e.g., *try and achieve*). If we take into account that the acquisition of logical relationships is a gradual process with the age of 8 years representing a milestone (Noveck, 2016), it is easy to understand why the Q implicatures can present certain difficulties in children with typical development. This type of implicature requires the consolidation of the logical relationships (specifically, negation, and entailment), which also fits the observations made by Pouscoulous et al. (2007), who stated that, when negation is explicitly introduced, the implicatures are more difficult to process. This occurs because, in the inferential process, a double negation takes place that leads to an affirmative element, and so the cognitive complexity is greater. From the statement “Not all of the students came” (or from “Some students did not come”), the child has to pragmatically infer that “Some students did come.”

In conclusion, the present study on the inferential capacity of GCI within the Levinson model in children with and without ASD has allowed us to advance beyond the commonly studied aspects of implicit language (e.g., metaphors, ironies, sarcasms, acts of indirect speech, jokes…). Furthermore, in the case of subjects with ASD, studies on the comprehension of GCI have generally been limited to scalar implicatures. In our study, we have extended the study of GCI to the other two types of implicatures, but in the case of the ASD group, we did not obtain evidence for an inferential continuum of the three types of GCI. Based on the data obtained in our study, we can conclude that children with level 2 ASD—of the chronological and linguistic age in our study- presented limitations in the comprehension of all three types of GCI.

### Limitations and future research

Our study presents some limitations that must be taken into account in interpreting and evaluating the reach of the results obtained, and it leads to proposals for possible future lines of research. First, children with ASD with very low cognitive functioning were not part of this sample, and so the autism spectrum was not entirely represented. In fact, it would be interesting to broaden this study to groups of children with level 1 and level 3 ASD. Specifically, in the children with level 1 ASD (high functioning), it would be interesting to find out if there are differences in the results obtained for the different types of implicatures, in order to explore the possibility of an inferential continuum of GCI, similar to the one proposed in this study for children with typical development. Second, this research did not include a comparison group with a different psychological disorder—e.g., ADHD-, and so we cannot definitively conclude that the group differences are unique to autism. Third, in the present study, the inferential continuum was constructed based on the degree of contextual dependence necessary to reach the inferential meaning. However, it cannot be assumed that the inferential process only takes this aspect into account as a factor. As Giora (1997) argued, the inferential process is a complex process in which different factors intervene (frequency, prototypicality, degree of ritualization/conventionalization …) that determine the greater or lesser difficulty of comprehending the type of inferential meaning in question. Undoubtedly, describing how these factors overlap when reaching a non-explicit meaning is a challenge that could open up a new perspective for future research in this field. Moreover, another proposal for future studies would be to carry out similar studies to this one with the rest of the non-literal meanings: the entailments, the presumptions, the conventional implicatures, and the PCI. It would be interesting to observe whether there is a gradual increase in errors as we move along the inferential continuum toward the more contextually dependent inferences, so that minimal errors could be expected on entailments, and more errors on PCI. Especially interesting would be the results obtained for the presumptions, a borderline category that lies between semantics and pragmatics (Levinson, 1983; Chierchia, 1995; Escandell-Vidal, 2005, 2006).

Finally, this research used cross-sectional data and did not study the variables over time. Thus, it would be advisable to carry out longitudinal studies that can explain how the different non-explicit meanings are acquired, in order to corroborate whether non-literal meanings are acquired according to the inferential continuum proposed: entailments would be acquired first, and PCI would be consolidated last. A longitudinal study would make it possible to find out whether, as hypothesized earlier, the acquisition of inferential skills is slower in ASD than in typical development during the childhood stage, and whether later, when stages of adolescence and adulthood are reached, these differences are diluted in most cases. In sum, a longitudinal study would provide valuable information about the evolution of the inferential processes over time in children with and without ASD.

## Author contributions

GP-C, JT, and M-IF-A: conceived and designed the work and constructed the GCI test and wrote the paper; GP-C, FG-S, and M-IF-A: acquired data; GP-C, FG-S, MM-A, and M-IF-A: corrected data; MM-A and M-IF-A: analyzed data; GP-C and JT: interpreted data; GP-C, JT, FG-S, and MM-A: drafted the article and revised it critically.

### Conflict of interest statement

The authors declare that the research was conducted in the absence of any commercial or financial relationships that could be construed as a potential conflict of interest.
