# Supplementary material for: Comprehension of Generalized Conversational Implicatures by Children With and Without Autism Spectrum Disorder
Source: Front Psychol. 2018 Mar 13;9:272. doi: 10.3389/fpsyg.2018.00272 (PMC5859064; doi:10.3389/fpsyg.2018.00272)
Supplement: Supplementary file 1 [file DataSheet1.docx]

APPENDIX 1. Generalized Conversational Implicatures test (GCI test).

| **Yesterday Juan tried to jump over the wall. (Q)** | | |
| --- | --- | --- |
| Juan did not manage to jump over the wall. | Juan did manage to jump over the wall. | Juan managed to stain his pants with mud. |
| **Some guests came to Maria’s party. (Q)** | | |
| Everyone Maria invited came. | Not all the guests came that Maria expected. | Exactly three guests came. |
| **Juan ate two candies. (Q)** | | |
| Juan ate two or more candies. | He ate four. | He ate exactly four candies and no more. |
| **Maria’s dress is red. (Q)** | | |
| Maria’s dress it red, but it could also be blue or green. | Maria’s dress is red and only red. | Maria’s dress has blood spots on it. |
| **It is possible that there is life on other planets. (Q)** | | |
| It is certain that there is life on other planets. | There may be life on other planets, but it’s not sure. | Someone saw a rabbit on Mars. |
| **If Juan does not do his homework, his mother punishes him. Juan’s mother punished him. (I)** | | |
| Juan’s mother punished him because he did something wrong. | Juan’s mother punished him because he didn’t do his homework. | Juan’s mother loves her son a lot. |
| **Maria and Juan, a happy married couple, bought a new flat. (I)** | | |
| Maria and Juan bought one flat to live in together. | Maria bought one flat, and Juan bought another one. | Juan wants to leave Maria and buy a flat far away from her. |
| **Yesterday we went to a very pretty restaurant but the food tasted very bad. (I)** | | |
| Yesterday we went to a restaurant and then we ate in another place where the food was very bad. | Yesterday we went to a restaurant, and in this restaurant, the food served was very bad. | Yesterday we went to a pizza parlor, and the pizzas they served us were delicious. |
| **Yesterday Anita went to the hospital, and the nurse gave her a shot. (I)** | | |
| A nurse on the street gave Anita a shot. | Anita went to a hospital, and there was a nurse who gave her a shot. | There aren’t any nurses in the hospital; there are only sick people. |
| **Manolito got hit very hard by a friend accidentally and left crying. (I)** | | |
| Manolito got hit very hard and, because of it, Manolito went away crying. | Manolito’s friend hit him, and so his friend went away crying. | Manolito and his friend hit each other, and they both cried. |
| **It is not impossible for the teacher to arrive late. (M)** | | |
| Although it doesn’t happen very often, the teacher could arrive late. | It is quite possible that the teacher will arrive late. | It is very common for the teacher to arrive late. |
| **Antonio made the car stop. (M)** | | |
| Antonio is a doctor who likes to stop cars. | Antonio put his foot on the brake of the car to stop it. | Antonio stopped the car, but in an unusual way: he did not put his foot on the brake of the car. |
| **Fernando went into the bar and a man laughed. (M)** | | |
| Fernando is a clown who works in a bar. | Fernando went into a bar, and someone who wasn’t Fernando laughed. | Fernando went into a bar and laughed as he entered. |
| **I saw a man play a musical instrument in the shape of a pipe that some people would call a flute. (M)** | | |
| There is a man playing the flute. | There is a man playing something similar to a flute. | There is a man who makes flutes and drums. |
| **Pedro’s neighbor caused Maria’s dog to die. (M)** | | |
| Pedro’s neighbor killed Maria’s dog himself. | Pedro’s neighbor made Maria’s dog die, but he did not kill it himself or not on purpose. | Pedro’s neighbor called the veterinarian to cure Maria’s dog. |

*Note: Type of implicature: Q, I, M.*
